# Antimutagenic activity and preventive effect of black tea on buccal mucosa cancer

**DOI:** 10.3892/ol.2013.1401

**Published:** 2013-06-14

**Authors:** YU QIAN, KAI ZHU, QIANG WANG, GUIJIE LI, XIN ZHAO

**Affiliations:** Department of Biological and Chemical Engineering, Chongqing University of Education, Chongqing 400067, P.R. China

**Keywords:** antimutagenicity, black tea, anticancer, buccal mucosa cancer, Kunming mice

## Abstract

A black tea product was evaluated for anti-mutagenic and *in vivo* anticancer effects. At concentrations of 1.25 and 2.5 mg/plate, black tea exhibited anti-mutagenicity with N-methyl-N′-nitro-N-nitrosoguanidine (MNNG) in the *Salmonella typhimurium* TA100 strain. A Kunming (KM) mouse buccal mucosa cancer model was established by injecting mice with U14 squamous cell carcinoma cells. Following injection, the wound at the injection site was smeared with black tea. It was observed that the tumor volumes for the groups treated with different concentrations of black tea were smaller than the control groups. The sections of buccal mucosa cancer tissue showed that cancer development in the black tea groups was weaker compared with that in the control group. Similar results were observed in the lesion section of the cervical lymph. Using reverse transcription polymerase chain reaction (RT-PCR), the black tea groups demonstrated an increase in Bcl-2-associated X protein (Bax) and a decrease in B cell lymphoma-2 (Bcl-2) expression, compared with the control groups. The results demonstrated that black tea had an improved antimutagenic effect and *in vivo* buccal mucosa cancer preventive activity compared with the untreated control in mice.

## Introduction

*Camellia sinensis* is the species of plant whose leaves and leaf buds are used to produce Chinese tea. White, green, oolong, pu-erh and black tea are all harvested from this species, but are processed differently to attain different levels of oxidation. Black tea is a completely oxidized tea. Black tea contains relatively high levels of polyphenolics with the major phenolics being the flavan-3-ols, the flavonols (mono-, di- and tri-glycoside conjugates of myricetin, quercetin and kaempferol), the flavones and the quinic acid esters of gallic, coumaric and caffeic acids (1). Black tea has a reduced flavan-3-ol monomer content and higher levels of polymerized derivatives, theaflavins, which account for ∼10–30% of the converted catechins and thearubigins (2,3). Although there is growing interest in the hypothesis that tea has a preventive effect against cardiovascular diseases and that tea polyphenols may mediate the observed benefits (4,5), the intricate mechanisms of polyphenolic action require further and comprehensive understanding.

Buccal mucosa cancer is the most common type of cancer of the oral cavity (6). The U14 mouse tumor is a squamous cell carcinoma, which was ectopically induced by treating the uterine cervix with 20-methylcholanthrene (7). After U14 cells were transplanted into mice, buccal mucosa cancer was induced (8). The present study aimed to determine the anti-mutagenic activities of black tea with N-methyl-N-nitro-N-nitrosoguanidine (MNNG) and to evaluate the cancer preventive effect of black tea using a mouse model of buccal mucosa cancer, in order to determine whether, as a functional food, black tea would demonstrate oral health benefits.

## Materials and methods

### Tea extract preparation

Qimen black tea (producer, Wuhu Huifu Tea Co., Ltd., Wuhu, China) was purchased from Anhui in China. To prepare the methanol extracts, black tea was freeze-dried and powdered. A ten-fold quantity of boiling water was added to the powdered sample and extracted twice by agitating. The water extract was evaporated using a rotary evaporator (N-1100; Eywla, Tokyo, Japan).

### Anti-mutagenic experiment

The *Salmonella typhimurium* strain, TA100, a histidine-requiring mutant bacterium, was maintained as described by Maron and Ames (9). In brief, 0.5 ml of phosphate buffer containing the direct mutagen of MNNG was distributed into sterilized capped tubes, and then 0.1 ml of test bacterial suspension from an overnight culture (1–2×10^9^ cells/ml) and 0.1 ml of test sample compound (50 *μ*l of mutagen and/or 50 *μ*l of test sample) were added. After agitating gently and pre-incubating at 37°C for 30 min, 2 ml of top agar, supplemented with L-histidine and D-biotin, kept at 45°C, was added to each tube and agitated for 3 sec. The entire resulting mixture was overlaid on a minimal agar plate. The plates were incubated at 37°C for 48 h and the revertant bacterial colonies on each plate were counted.

### Animals

Seven-week-old female Kunming (KM) mice were purchased from the Experimental Animal Center of Chongqing Medical University (Chongqing, China). The animals were maintained in a temperature-controlled (23±1°C; relative humidity, 50±5%) facility with a 12-h light/dark cycle and had unlimited access to a standard mouse chow diet and water. The protocol for these experiments was approved by the Animal Ethics Committee of Chongqing Medical University (Chongqing, China).

### Cell preparation

U14 squamous cell carcinoma cells obtained from the Chinese Academy of Medical Sciences (Beijing, China) were used in this study. The cancer cells were cultured in RPMI-1640 medium (Gibco Co., Birmingham, MI, USA) supplemented with 10% fetal bovine serum (FBS) and 1% penicillin-streptomycin (Gibco-BRL, Grand Island, NY, USA) at 37°C in a humidified atmosphere with 5% CO_2_ (incubator model, 311 S/N29035; Forma, Waltham, MA, USA). The medium was changed 2 or 3 times a week (10). *In vitro* cultured U14 cells (5×10^6^/mouse) were injected into the abdominal cavity of the 7-week-old female KM mice. After 1 week, the carcinoma ascites were collected and diluted in sterile saline to a concentration of 1×10^7^/ml.

### Induction of buccal mucosa cancer

To investigate the preventive effects of the black tea against buccal mucosa cancer induced by injecting U14 cells into the mice, the animals were divided into 4 groups with 10 mice in each. The experimental design was as follows; the mice in the black tea sample groups were smeared with 0.2 ml black tea solution (200 or 100 mg/ml) onto the buccal mucosa every 12 h for 14 days. The control and black tea sample groups were then inoculated with 0.05 ml cancer cell suspension (1×10^7^/ml) on the buccal mucosa. The black tea samples continued to be smeared on the buccal mucosa of the mice every 12 h. The normal group were not treated with the cancer cell suspension. The mice were sacrificed 14 days later and their tumor volumes and lymph node metastasis rates were determined (8).

### Histological grading of buccal mucosa cancer

Buccal mucosa tissues were removed and embedded into paraffin for histological analysis using hematoxylin and eosin (HE) staining. Buccal mucosa cancer was graded as follows: i) well-differentiated carcinoma, cells resembling the adjacent benign squamous epithelium; ii) moderately-differentiated carcinoma, cells forming large anastomosing areas in which keratin pearls are formed, they are not numerous and the main component consists of cells with pronounced cytonuclear atypia; and iii) poorly-differentiated carcinoma, cells that have lost the majority of their squamous epithelial characteristics and architecture (11).

### Reverse transcription polymerase chain reaction (RT-PCR) analysis of Bcl-2-associated X protein (Bax) and B cell lymphoma-2 (Bcl-2) mRNA expression

Total RNA was isolated using TRIzol reagent (Invitrogen, Carlsbad, CA, USA) according to the manufacturer’s instructions. RNA was digested with RNase-free DNase (Roche, Basel, Switzerland) for 15 min at 37°C and purified using an RNeasy kit (Qiagen, Hilden, Germany) according to the manufacturer’s instructions. cDNA was synthesized from 2 *μ*g total RNA by incubation at 37°C for l h with avian myeloblastosis virus (AMV) reverse transcriptase (GE Healthcare, Uppsala, Sweden) with random hexanucleotides, according to the manufacturer’s instructions. The sequences of the primers used to specifically amplify the genes of interest are shown in Table I. Amplification was performed in a thermal cycler (Eppendorf, Hamburg, Germany) with 29 Bax cycles, 34 Bcl-2 cycles and 25 GAPDH cycles of denaturation. The amplified PCR products were run on 1.0% agarose gels and visualized by ethidium bromide (EtBr) staining (12).

### Statistical analysis

Data are presented as the mean ± standard deviation. Differences between the mean values for the individual groups were assessed using a one-way analysis of variance (ANOVA) with Duncan’s multiple range tests. P<0.05 was considered to indicate a statistically significant difference. The SAS v9.1 statistical software package (SAS Institute Inc., Cary, NC, USA) was used for the analysis.

## Results

### Antimutagenic effects of black tea

The black tea demonstrated inhibitory effects on spontaneous mutations in the *Salmonella typhimurium* TA100 strain (Table II). At 1.25 mg/plate, the spontaneous mutation inhibitory rate of black tea was 57%. At 2.5 mg/plate, black tea revealed an inhibition rate of 79%. These results indicate that the black tea exerted a decreasing effect on the spontaneous levels of mutation.

Black tea showed an anti-mutagenic effect in the *Salmonella typhimurium* TA100 strain when treated with MNNG (Table III). At 1.25 mg/plate, the mutagenic inhibition rate of black tea was 32%, demonstrating an anti-mutagenic effect. When the black tea concentration was 2.5 mg/plate, black tea further showed significantly increased anti-mutagenic effects, with an inhibition rate of 63%. This suggested that black tea had a strong anti-mutagenic effect.

### Tumor volumes and lymph node metastasis rates

Buccal mucosa cancer was induced by injecting U14 cells into mice. After 14 days, the mice in all groups presented with carcinogenesis. The tumor volumes of the buccal mucosa tissues were measured. The tumor volumes for the control, 100 mg/ml black tea and 200 mg/ml black tea groups were 10.8, 9.7 and 5.2 mm^3^, respectively (Table IV). There were 6 mice demonstrating lymph node metastasis in the control group, 4 in the black tea (100 mg/ml) group and 1 in the black tea (200 mg/ml) group. Consequently, the lymph node metastasis rate was 60, 40 and 10%, respectively. These results demonstrate that black tea is effective in impeding carcinogenesis, proliferation and metastasis.

### Histopathology of buccal mucosa tissues

**Figure 1.**
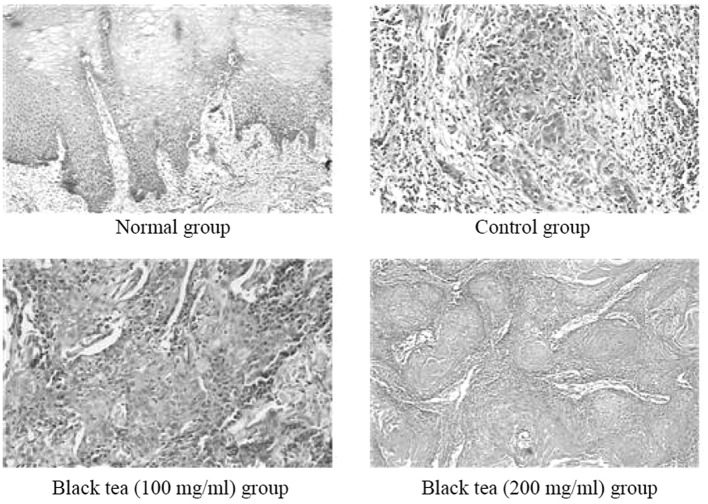
Histology of buccal mucosa tissues induced by injecting U14 squamous cell carcinoma cells into mice (hematoxylin and eosin staining; magnification, ×100).

Histological changes in the buccal mucosa of mice injected with U14 cells were examined by HE staining. The histological tissue sections of the mice in the normal group demonstrated a normal histological morphology for squamous epithelial tissue. The histopathological evaluation revealed indications of buccal mucosa cancer in the two groups administered with U14 cells (Fig. 1). The sections from the mice in the control group revealed that the tissue had lost its squamous epithelial characteristics and architecture (grade iii). The tissue sections of the black tea (100 mg/ml) group looked less like normal squamous epithelium (grade ii). The tumor cells remained in nests but there were few larger, eosinophilic, polygonal cells that were trying to layer themselves in a squamous-like fashion. The tissue sections of the black tea (200 mg/ml) group appeared much like the adjacent benign squamous epithelium (grade i). From these sections, it was demonstrated that black tea provided a preventive effect against buccal mucosa cancer.

### Gene expression of Bax and Bcl-2 in buccal mucosa tissues

**Figure 2.**
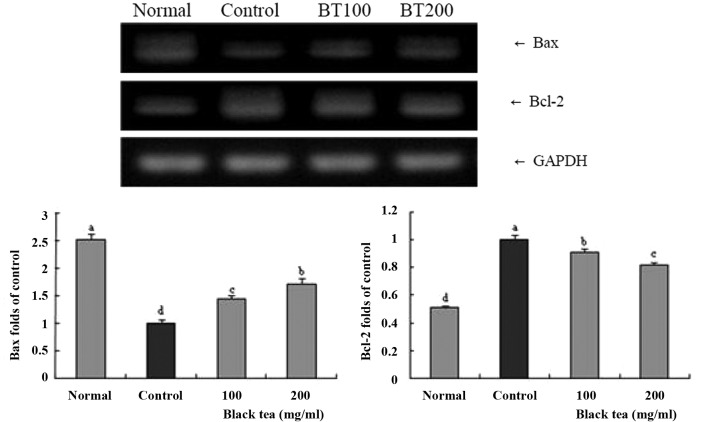
Effects of black tea sample on mRNA expression of Bax and Bcl-2 in buccal tissues. Upper panel, western blot analysis of the expression; lower panel, quantitation of the expression. Band intensity was measured with a densitometer and expressed as a fold-rate of the control. Fold ratio = gene expression / xGAPDH x control numerical value (control fold ratio, 1). ^a–d^Mean values with different letters over the bars are significantly different (P<0.05) according to Duncan’s multiple range test. BT100, black tea (100 mg/ml); BT200, black tea (200 mg/ml); Bax, Bcl-2-associated X protein; Bcl-2, B cell lymphoma-2.

To determine the protective mechanisms against buccal mucosa cancer, the expression of the Bax and Bcl-2 genes in the buccal mucosa tissues was determined by RT-PCR. As shown in Fig. 2, in the group treated with black tea (100 mg/ml), significant changes were demonstrated in the pro-apoptotic activities of Bax and the anti-apoptotic activities of Bcl-2. Accordingly, the results suggest that the black tea induced apoptosis in buccal mucosa tissues via a Bax- and a Bcl-2-dependent pathway. Thus, apoptosis induction by the black tea (20 mg/ml) group was related to an increase in Bax and a decrease in Bcl-2 in terms of mRNA and protein expression compared to black tea (100 mg/ml) and control groups. From the results, it was observed that black tea showed a good buccal mucosa cancer protective effect.

## Discussion

Tea has numerous functional compounds, including polyphenols, catechins, amino acids and vitamins (13). Studies have demonstrated that there are a number of important compounds to aid in the prevention of cancer (14,15). Green tea extract has the highest amount of epigallocatechin gallate (EGCG) and EGC among the four extracts (green, Oolong, black and Pu-erh tea). However, EGCG and EGC have not been detected in Pu-erh tea. The features of fermented tea (Oolong, black and Pu-erh tea) greatly differ from those of green tea, which is non-fermented and is preferably drunk as fresh as possible (16).

The various health benefits of black tea have made it become known as the medicinal tea plant. Pasha *et al* (17) proposed that the intake of fermented tea is superior to black tea in terms of its nutritive and therapeutic value; it also does not show much change in taste and color after fermentation. This may be recommended for consumption as a modified beverage with higher nutritive and medicinal values.

Deleterious mutations produced by mutagens in the DNA may result in aberrant, impaired or lost function in a particular gene, and accumulation of such mutations may lead to cancer (18). The Ames test is a rapid test that is able to screen for chemical carcinogens. Biological genetic mutation is regarded as a causative key factor of cancer (19), and *Salmonella typhimurium* is used as a testing strain in a biological assay to assess the mutagenic potential of chemical compounds (20).

Apoptosis induction in cancer cells is initially identified by morphological changes including cell shrinkage, membrane blebbing, chromatin condensation and nuclear fragmentation (21). Apoptosis is an important defense against cancer. This process involves the elimination of potentially harmful cells. Numerous diseases have been associated with dysregulated apoptotic processes, ultimately leading to the inhibition of cell death and propagation of diseases such as cancer. Elucidating the critical events associated with carcinogenesis provides an opportunity for preventing cancer development via dietary intervention by inducing apoptosis, particularly with bioactive agents or functional foods. Diet and drink are significant environmental factor in the overall cancer process, and may exacerbate or interfere with disease progression. In addition to dietary effects on protein expression and function, evidence is also accumulating that a large number of food components may exert effects on the human genome, by either directly or indirectly modulating gene expression (22,23). Bax, a protein that is a member of the Bcl-2 gene family, promotes apoptosis by competing with Bcl-2 (24). In a healthy cell, the anti-apoptotic protein Bcl-2 is expressed on the outer mitochondrial membrane surface (25). As Bax and Bcl-2 genes are mainly expressed during apoptosis, we determined that these genes regulate apoptotic activity.

Histopathology is an important tool in anatomical pathology, since the accurate diagnosis of cancer usually requires a histopathological examination of the samples. Histopathology is a clinical standard for the diagnosis of oral cancer (26). Histopathological assays permit the precise determination of the anti-cancer effects of black tea.

In summary, the present study employed *in vitro* and *in vivo* experimental methods, such as the Ames test and histopathology and RT-PCR analyses, to evaluate the anti-cancer effects of black tea. The results from the present study demonstrate that black tea may decrease the spontaneous revertants in *Salmonella typhimurium* TA100 and may also decrease the mutagenic effect of MNNG. A mouse model bearing tumors produced by squamous cell carcinoma U14 cells was used to study the *in vivo* effects of black tea. The results showed that black tea had strong anti-cancer activities against buccal mucosa cancer. Overall, black tea demonstrated *in vitro* anti-mutagenic effects and *in vivo* anti-cancer and anti-metastatic activities. In conclusion, an increased concentration may be used to increase the oral cancer preventive effect of black tea.

## Figures and Tables

**Table I.** Sequences of RT-PCR primers used in this study.

Gene name	Sequence
Bax	Forward: 5′-AAG CTG AGC GAG TGT CTC CGG CG-3′
	Reverse: 5′-CAG ATG CCG GTT CAG GTA CTC AGT C-3′
Bcl-2	Forward: 5′-CTC GTC GCT ACC GTC GTG ACT TGG-3′
	Reverse: 5′-CAG ATG CCG GTT CAG GTA CTC AGT C-3′
GAPDH	Forward: 5′-CGG AGT CAA CGG ATT TGG TC-3′
	Reverse: 5′-AGC CTT CTC CAT GGT CGT GA-3′

RT-PCR, reverse transcription polymerase chain reaction; Bax, Bcl-2-associated X protein; Bcl-2, B cell lymphoma-2.

**Table II.** Effect of black tea on spontaneous mutagenicity.

Treatment	No. of revertants/plate	
	1.25 mg/plate	2.5 mg/plate
Spontaneous (No Mutation)	128±15^a^	
Black tea	55±9^b^ (57)	27±7^c^ (79)

Values are the mean ± SD of revertants/plate. Values in parentheses are the inhibition rates (%).

a–c Mean values with different letters in the same column are significantly different (P<0.05) according to Duncan’s multiple range test.

**Table III.** Effect of black tea on the mutagenicity induced by MNNG (0.4 *μ*g/plate) in *Salmonella typhimurium* TA100.

Treatment	No. of revertants/plate	
	1.25 mg/plate	2.5 mg/plate
Spontaneous (No mutation)	128±15	
MNNG (control)	922±37^a^	
Black tea	668±22^b^ (32)	422±24^c^ (63)

Values are the mean ± SD of revertants/plate. Values in parentheses are the inhibition rates (%) calculated as: [(MNNG - spontaneous) - (black tea - spontaneous)] / MNNG - spontaneous.

a–c Mean values with different letters in the same column are significantly different (P<0.05) according to Duncan’s multiple range test. MNNG, N-methyl-N-nitro-N-nitrosoguanidine,

**Table IV.** Tumor sizes and lymph node metastasis rates of black tea sample smeared on mice.

	Normal group	Control group	Black tea groups	
			100 mg/ml	200 mg/ml
Tumor volume (mm^3^)	0	10.8±0.6^a^	9.7±0.5^b^	5.2±0.2^c^
Lymph node metastasis^d^	0	6/10 (60%)	4/10 (40%)	1/10 (10%)

a–c Mean values with different letters in the same column are significantly different (P<0.05) according to Duncan’s multiple range test.

d Number of lymph node metastasis/total number.
